# Much more than a biological phenomenon: A qualitative study of women’s experiences of brain fog across their reproductive journey

**DOI:** 10.1177/13591053241290656

**Published:** 2024-10-25

**Authors:** Hannah Johnson, Jane Ogden

**Affiliations:** University of Surrey, UK

**Keywords:** brain fog, experiences, hormones, identity, reproductive journey

## Abstract

Whilst ‘brain fog’ is mostly considered a biological problem little is understood about an individual’s experience. This qualitative study explored women’s experiences of brain fog focusing on those at the start (aged 18–25; *n* = 10) and end (aged 45–60; *n* = 10) of their reproductive journey. Descriptive thematic analysis described three themes: (i) ‘daily disruptions’ describing cognitive dysfunctions and the main triggers; (ii) ‘the cycle of impact’ with a focus on women’s emotional experiences and how these can exacerbate brain fog; (iii) ‘taking control’ highlighting the use of self-care, physical prompts and Hormonal Replacement Therapy (HRT) to manage brain fog. Transcending these themes was the notion of ‘crisis of identity’ illustrating the negative impact of brain fog on the women’s sense of self with some older women describing acceptance and finding it less challenging. Brain fog is much more than a biological phenomenon and has broader implications for a woman’s sense of self.

The term ‘brain fog’ is commonly used colloquially to describe temporary experiences of forgetfulness, cloudiness, difficulty with thinking or processing information, as well as trouble with learning, speaking or understanding (Callan et al., 2022; Ross et al., 2013). These impairments may affect an individual’s daily tasks or working, as well as impeding their decision-making, communication and social relationships (Callan et al., 2022) and can be associated with lower work functioning and ultimately a poorer quality of life (Asadi-Pooya et al., 2022). Whilst brain fog is not recognised as a medical condition (Sukel, 2022), it is commonly considered within neurocognitive disorders (NCDs) or mild cognitive impairments (MCI), which are defined as a transitionary condition between typical cognitive behaviour and dementia (Petersen et al., 2009). Brain fog appears to be both complex and variable with no two individuals having the same experience (Sukel, 2022). Furthermore, the duration of the difficulties associated with brain fog varies with some suffering episodes for as little as a day, to others experiencing it for weeks or years (Sukel, 2022). This therefore makes brain fog difficult to diagnose and treat. To date, much recent research has explored brain fog specifically within the domains of Long COVID, postural tachycardia syndrome (PoTS) and the menopause.

Since the COVID-19 global pandemic, brain fog has gained a surge in research due to its prevalence as a possible symptom of Long COVID syndrome (LCS). In a large cohort study of 2696 adult patients (aged 18–55) surviving COVID-19, Asadi-Pooya et al. (2022) investigated brain fog as one of the long-term side effects of the disease. In total, 1680 patients reported chronic symptoms of LCS, of which 194 (7.2%) patients reported brain fog as a common long-term side effect, which was more prevalent in women (Asadi-Pooya et al., 2022). Due to the infection from COVID-19, and its possible alterations to brain functioning, the research concluded that the LCS, such as brain fog, may be a biological phenomenon (Asadi-Pooya et al., 2022). In line with this, Callan et al. (2022) carried out a large-scale qualitative study (*n* = 50) to explore the experiences of those suffering from ‘brain fog’ as part of Long Covid. The results provided a rich insight into patients’ experiences with themes such as whether ‘brain fog’ was the most appropriate term to use, how their symptoms fluctuated over time, the wide-ranging impact of their experiences on factors such as relationships and identity, feelings of guilt, shame and stigma and the challenges they faced trying to find help. Since the Covid pandemic several similar qualitative studies have been conducted exploring the symptoms of Long Covid and many have similarly addressed the experiences of brain fog in this population (e.g. Chasco et al., 2022; Heiberg et al., 2022; Rofail et al., 2024).

In a similar vein, Ross et al. (2013) explored the symptoms of brain fog as a side effect of postural tachycardia syndrome (PoTS). PoTS is a chronic form of orthostatic intolerance defined by the onset of orthostatic symptoms associated with an increase in heart rate after getting up from sitting or lying down. In a sample of 138 PoTS patients, aged 14–29 (88% female), brain fog was assessed via a cognitive symptom questionnaire consisting of 38 questions, specifically designed for this study (Ross et al., 2013) together with the Wood Mental Fatigue Inventory (Ross et al., 2013). The findings showed that brain fog was experienced daily for 67% of participants, and was most often triggered by physical fatigue, lack of sleep, prolonged standing and dehydration. Other general patterns of brain fog were assessed, noting the top descriptors being forgetful, difficulty thinking, focusing and communicating (Ross et al., 2013).

Research indicates that brain fog also commonly occurs during a woman’s menopausal transition, also termed perimenopause (Greendale et al., 2009, 2010, 2020; Sherman, 2005; Woods and Mitchell, 2011; Weber et al., 2012, 2014). Menopause typically occurs between the ages of 45 and 55 years, with many women experiencing years of inconsistent ovarian decline and erratic production of oestrogen and progesterone (Ledford, 2023). In a cross-sectional analysis of a population-based cohort (*n* = 230), Sullivan Mitchell and Fugate Woods (2001) reported that 62% of women suffer from subjective cognitive problems during their menopausal transition; the symptoms included difficulty retrieving words or numbers, forgetting the purpose of a behaviour, losing their train of thought and overlooking appointments. Likewise, women who are peri-menopausal have been found to report greater subjective cognitive difficulties than pre-menopausal or post-menopausal women (Weber et al., 2014) and research from a large cohort (*n* = 2000) indicated that decrements in cognitive processing speed, verbal encoding and episodic memory experiences during perimenopause mostly resolve by the post-menopausal period (Greendale et al., 2009, 2010). Furthermore, associations have been found between menopausal related perceived memory problems and depression, anxiety, somatic symptoms and sleep disturbance (Weber et al., 2012) and with work and relationships (Woods and Mitchell, 2011).

Such research therefore suggests that brain fog may be a biological phenomenon (Asadi-Pooya et al., 2022) that disproportionately affects women (Asadi-Pooya et al., 2022; Greendale et al., 2020; Ross et al., 2013), is influenced by hormones (Greendale et al., 2020), is a core part of the menopausal transition (Greendale et al., 2009, 2010; Weber et al., 2012) and maybe a comorbidity of health conditions such as PoTS (Ross et al., 2013) and Long Covid (Callan et al., 2022). Research also indicates, however, that brain fog may be modified by diet and lifestyle (Shrividya and Joy, 2021). Focusing on the diet and occupation of 100 perimenopausal females, comparative research by Shrividya and Joy (2021) assessed the cognitive functioning of lacto non-vegetarian, lacto-vegetarian, homemakers and employed women, using the MMSE. The results concluded no significant difference between lacto-vegetarian homemakers and employed lacto-vegetarian females. However, employed lacto-non-vegetarian women had better cognitive function than lacto-non-vegetarian homemakers, whilst lacto-vegetarian women had better cognitive functioning than the lacto-non-vegetarian women (Shrividya and Joy, 2021). This means that across all perimenopausal women, lacto-non-vegetarian homemakers had the poorest cognition. Whilst brain fog may therefore relate to hormones, both diet and lifestyle can modify brain fog experiences during the menopause transition.

In summary, whilst research exploring brain fog is limited, existing studies indicate that brain fog is probably a biological phenomenon, found predominantly within females, which is influenced by hormone changes, illness and modified by diet and lifestyle. To date, however, whilst some qualitative research has explored brain fog as a symptom of Long Covid (e.g. Callan et al., 2022; Heiberg et al., 2022; Rofail et al., 2024) less is known as to how women experience brain fog as their hormones change across their reproductive journey and when they may take hormones to manage their fertility and menopause which can involve the addition of hormones (i.e. the contraceptive pill) or the replacement of hormones as they decline (i.e. HRT). For the purpose of this study, the notion of a reproductive journey reflected the years of a woman’s life when she would be either considering having children and/or being impacted by the changes induced by the menopause indicating that this time was over (18–60 years).

The present qualitative study therefore aimed to explore women’s experiences of brain fog across their reproductive journey, using two different age groups: younger women aged 18–25 years (start of their reproductive journey), and older women aged 45–60 years (end of their reproductive journey) in the context of changing hormones at different life stages. Furthermore, this study also aimed to explore the potential role of hormonal medication, such as the contraceptive pill or hormone replacement therapy (HRT), on experiences of brain fog.

## Method

### Design

This study used qualitative methods with in-depth semi-structured interviews to explore the experiences of brain fog in women in two age groups: 18–25 years and 45–60 years, to reflect either end of the reproductive pathway. All data was analysed by descriptive thematic analysis (TA) (Clarke and Braun, 2017). Favourable ethical approval was obtained from the University Ethics Committee, FHMS 23-24 002 EGA. All participants gave written consent prior to taking part in the study.

### Sample

Sixty two female participants completed an initial screening survey, with 27 individuals volunteering an email address for the follow-up interviews. Inclusion criteria were: female, aged between 18 and 25 or 45 and 60 years, have experienced brain fog, be English speaking and living in the UK. Of these, 20 women who had experienced brain fog took part in the interviews (young female group, *n* = 10; older female group, *n* = 10). Participant demographics with pseudonyms are shown in Table 1.

**Table 1.** Participant demographics.

Group 18–25 years				Group 45–60 years			
Pseudo	Age	Contraceptive pill?	Ethnicity	Pseudo	Age	HRT?	Ethnicity
Cora	22	Previously	White British	Dorothy	54	Yes	White British
Dolly	21	Previously	White British	Jane	52	No	White British
Grace	22	Previously	White British	Mary	52	Previously	White British
Penelope	21	Previously	White British	Georgia	49	No	White British
Darcey	20	Yes	White British	Maria	52	No	Black/African American
Lucy	23	Previously	White British	Melissa	55	Yes	White British
Charlotte	21	Never	Black African	Catarina	48	Yes	White British
Josephine	19	Never	White British	Margaret	53	Yes	White British
Bryony	18	Never	White/Black Caribbean	Sophie	47	Yes	White British
Louisa	22	Never	White British	Hannah	50	Yes	White British

HRT: hormone replacement therapy.

### Data collection

#### Screening survey

The Qualtrics survey included general demographic questions (age, ethnicity), whether they had experienced brain fog and whether they took the contraceptive pill or HRT. Participants were invited to take part if they had ever experienced brain fog which was defined as ‘difficulty thinking or concentrating’.

#### Interview schedule

The interview began with a quick briefing on the topic of brain fog and the participants were reminded of the working definition of brain fog as ‘difficulty thinking or concentrating’. The interview schedule consisted of the following questions with prompts: ‘Have you ever experienced brain fog?’ (how and when did it occur?; what made it better or worse?; how did this make you feel?); ‘How do you think this may have changed across your lifetime?’ (what factors affected your experience of brain fog?); ‘Do you currently take either the contraceptive pill or HRT?’ (has this affected your experience of brain fog?).

### Procedure

Participants were recruited through social media, the University Research Portal and snowballing. After the signed consent form had been returned to the researcher, a Microsoft Teams meeting was scheduled at a mutually convenient time. Interviews lasted between 10 and 25 minutes with a median time of 19 minutes. Using the Microsoft Teams transcription as a basis, each transcript was thoroughly checked against the audio recording to ensure accuracy. Interviews were anonymised using pseudonyms.

### Data analysis

Data was analysed using inductive Descriptive Thematic Analysis (Braun and Clarke, 2017). The following steps were taken: familiarisation with the data set by reading and re-reading the transcripts several times; assignment of codes to summarise items of interest; identification of overlapping or related codes; re-examination of codes to refine themes; re-reading data to check consistency of themes; and finally, defining themes and translating them into a meaningful and unified narrative. In keeping with the reflexive element of thematic analysis, reflective discussions within the research team were ongoing throughout the process and the researcher kept a reflexive diary.

### Positionality

This research was completed by two women aged matched to the two groups of focus. The younger female researcher carried out all the interviews and was aged 22. She had previously experienced brain fog whilst taking the contraceptive pill. The older women was aged 58, had not experienced brain fog but was taking HRT. These different perspectives and expectations were discussed throughout the research process as a means to promote a degree of distance from the data. Both researchers were psychologists by discipline. The older researcher was a senior health psychology professor. The younger researcher was completing her psychology degree.

## Results

**Figure 1.**
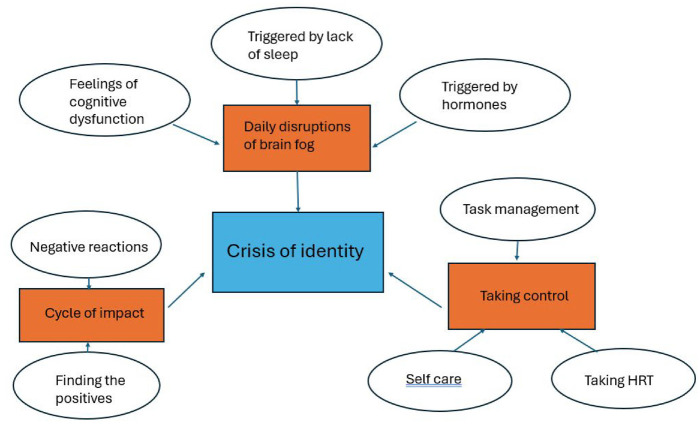
Thematic map.

Analysis of the data described three major themes, each with subthemes: ‘Daily Disruptions of Brain Fog’; ‘The Cycle of Impact’ and ‘Taking Control’. Transcending these themes was the notion of a ‘Crisis of Identity’, whereby participants questioned their self-worth and self-belief due to their diminished capabilities because of experiencing brain fog. These themes are illustrated in a thematic map (Figure 1) and will be described and illustrated with exemplar quotes. The codes ‘Y’ and ‘O’ are used to denote which participants are the younger females (18–25 years) and those that are the older females (45–60 years).

### Theme 1: Daily disruptions of brain fog

Participants highlighted how brain fog was experienced as cognitive dysfunction, which disrupted their daily living and was often triggered by hormones and a lack of sleep.

#### Feelings of cognitive dysfunction

Women from both age groups described how their daily lives were disrupted by feelings of cognitive dysfunction including difficulty concentrating and having comprehensive thoughts. For example, both Mary (O) and Cora (Y) reported similar experiences:

You’re not in a good state to take things in and comprehend things. (Mary).

I’ll have days where I have trouble concentrating and nothing goes in my brain. (Cora).

For the older participants, however, the emphasis was also on feeling increasingly disorganised. For example, Maria (O) found it difficult to organise her thoughts and would often question whether she knew what she was talking about:

You might be in a meeting, and you’ll be talking about something, and you’ll be like

“I think I might’ve just lost track, like do I even know what I am talking about?”…

Brain fog, for me, is difficulty managing the mental load. (Maria).

In contrast, the younger participants tended to describe their cognitive dysfunction as needing increased effort to function normally. For example, Grace (Y) expressed that comprehensive thoughts are a lot more effort than they should be when she experiences brain fog, whilst Louisa (Y) said:

When in conversation with people, it takes additional effort to think and communicate and do things that don’t require that much effort. (Louisa).

Therefore, despite the slight difference in the descriptions of their brain fog, both age groups experienced significant cognitive dysfunction that impacted their daily lives due to an increased struggle to function.

#### Triggered by a change in hormones

Women also described the triggers to their symptoms of brain fog with a focus on a change in their hormones. For many of the older participants, their brain fog started at the same time as their perimenopause. Moreover, they also recognised that the hormones associated with the menopause disrupted their sleep, exacerbating their brain fog: For example, Margaret (O) explained:

Menopause impacts your sleep, so it’s a vicious cycle of lack of sleep making the brain fog worse (Margaret).

In contrast, the younger women attributed their brain fog to their hormones during their menstrual cycle. For example, Penelope (Y) said:

Around the time of my period, I feel particularly drained in energy, which I notice causes my brain fog … I am more in touch with how I am feeling and so probably recognise it more (Penelope).

Furthermore, some of the younger participants highlighted that the hormones associated with their contraceptive pill triggered their brain fog. Both Grace (Y) and Darcey (Y) reported similar experiences:

When I stopped taking the birth control (contraceptive) pill, my brain fog eased up a bit … It was a lot worse when I was on the pill versus now (Grace).

It was a similar time I started to get more brain fog, when I started on the

[contraceptive] pill (Darcey).

Whilst Lucy (Y) also confirmed the hormonal influence triggered her cognitive dysfunction associated with brain fog:

My mental and overall clarity is lacked more severely when I’m on the

[contraceptive] pill (Lucy).

Therefore, across both age groups, all participants expressed how their hormones affected their energy levels, through lack of sleep or due to feeling drained, which ultimately exacerbated their cognitive dysfunctions, increasing episodes of brain fog.

#### Triggered by a lack of sleep

In addition, a further trigger to brain fog for both age groups was the lack of sleep and subsequent tiredness. For example, Jane (O) and Charlotte (Y) shared similar experiences:

Tiredness is your worst enemy, it increases anxiety and increases your inability to function. (Jane).

If I sleep less, it makes it worse (Charlotte).

Whilst Hannah (O) and Darcey (Y) expressed how their tiredness associated with their working weeks triggers their brain fog:

By the end of the week, Thursday and Friday, I’m struggling because of tiredness, and it will last until I rest (Hannah).

If I am particularly burnt out, it’s more likely to happen … so towards the end of the semester, maybe the last couple of weeks I will feel more tired and overworked, so it becomes harder to concentrate and harder to keep going (Darcey).

Sleep was therefore key for all participants but the time frame seemed different with younger women focusing on weeks and the older women considering changes over days.

In summary, this theme illustrates how brain fog was experienced as a sense of cognitive dysfunction, with both age groups highlighting changes in hormones or a lack of sleep as key triggers.

### Theme 2: The cycle of impact

Participants described how their brain fog made them feel, in terms of stress and anxiety or managing to find the positives in their experiences. This was further reflected in a cycle of impact whereby worrying about having brain fog exacerbated these experiences.

#### Negative reactions

Participants from both age groups described a range of negative reactions when discussing how they felt about their brain fog. Frequently, these emotions exacerbated the frequency of brain fog, which ultimately made them question their self-worth and capabilities, creating a cycle of impact. For example, Dolly (Y), Josephine (Y) and Hannah (O) shared their experiences of feeling stressed because of their brain fog, which created a negative cycle of impact, as it caused their brain fog to increase:

I am conscious when I am talking and I am making no sense, then I get stressed and end up making less sense, so I stop talking because I don’t have a clue what I am on about (Dolly).

It’s really stressful. I feel the more you think about it, the more it happens (Josephine).

If I get stressed by it, it’ll make it worse (Hannah).

Similarly, Mary (O) discussed this negative cycle of impact in relation to feelings of anxiety:

You get that lapse of concentration, which makes you anxious, and then your brain fog gets worse (Mary).

Thus, feeling negative emotions towards experiences of brain fog ultimately creates a negative cycle of impact, worsening its symptoms.

#### Finding the positives

In contrast, however, some older women also managed to find some positives in their experienced and reframed their brain fog in a positive way. For example, Georgia (O) embodied her brain fog without embarrassment, and Dorothy (O) was positive that it would not last forever:

I’m just going with it, just kind of accepting it for the moment … I don’t want to make it the focus of that defines me, so I embrace it as part of me (Georgia).

I feel positive I will get through it because I have older friends that no longer have brain fog (Dorothy).

Similarly, Maria (O) described how she understood how the cycle of impact brain fog can be exacerbated by her negative emotions, so remained positive by not allowing it to bother her:

I care less, because you can’t afford to stress about it … if you continue to stress, the brain fog will be so much worse (Maria, O)

Overall, this theme illustrates the cycle of impact caused by experiencing brain fog, and how this commonly occurs because of the stress and negative emotions attached with the phenomenon. Whilst both age groups described their emotions negatively, and explained how this created a cycle of impact, it was only the older female age group that expressed positive responses to their brain fog and managed to reframe it in a more beneficial way.

### Theme 3: Taking control

Participants explained how they took control of their brain fog by self-care, task management or by taking Hormone Replacement Therapy (HRT).

#### Self-care

For both age groups, one method of taking control of their brain fog was self-care which for all participants involved basic lifestyle management such as sleep, diet and exercise. For the younger females such forms of lifestyle management tended to be their biggest forms of self-care. For example, Darcey (Y) and Grace (Y) shared similar methods of taking control:

Sleep is a big one for my concentration, and making sure I am eating enough of the right things (Darcey).

Reset overnight, have a big breakfast in the morning, and I am good to go (Grace).

In contrast, whilst the older females also managed their lifestyles, they were more likely to emphasise prioritising themselves as their form of self care. For example, Maria (O) highlighted the importance of being respectful to herself:

Not being mindful to yourself is a hindrance, so my brain fog will be affected by me not being kind to myself (Maria).

Whilst Jane (O) expressed her taking control as focusing on her health:

I would do things that calmed me, then I could keep going. I also make sure to take vitamins to give me energy, and when I feel energised, it gets rid of my brain fog (Jane).

Therefore, despite the slight differences in behaviours, both female age groups expressed self-care as a means to take control of their brain fog.

#### Task management

Participants also described using strategies to help them manage their tasks as a method to take control of their brain fog. This technique was found across all interviews regardless of age group. For example, both Catarina (O) and Bryony (Y) reported similar experiences:

I must sit down, write lists, and decide what I’m going to do first and tackle one thing at a time. Otherwise, if I try to do these things at once it confuses my brain. (Catarina).

In the morning, when I struggle to know what to do next, I get a pen and paper and write everything down in a list and organise it that way. (Bryony).

Thus, both female age groups took control of their brain fog in a similar manner by using task management strategies to deal with the associated cognitive dysfunction.

#### Using HRT

Finally, the older participants also described how taking HRT was a valuable method of taking control of their brain fog. For example, Mary (O), Sophie (O) and Melissa (O) agreed that their brain fog had improved since taking HRT. For example, Melissa (O) said:

HRT has helped. I wouldn’t say it’s gone away completely, but I definitely think I get it less and I’m more stable (Melissa).

Whilst Dorothy (O) expressed feeling more in control:

I feel a lot less ‘lost’ since being on HRT (Dorothy).

In summary, this theme encapsulates how participants took control of their brain fog. When focusing on self-care, the younger females would prioritise their sleep and eating well, whilst the older females highlighted the importance of being mindful of their needs and capabilities and using HRT. However, both age groups use task management strategies as a means to take control of their experiences.

### Transcending theme: Crisis of identity

Participants therefore described their brain fog in terms of the daily disruptions, its cycle of impact and how they took control. Transcending these themes was the notion of a ‘crisis of identity’, reflecting how brain fog caused participants to question their self-belief, self-worth and who they were.

For example, participants expressed how changes in their hormone often triggered the daily disruptions associated with brain fog, which for some, created a negative cycle of impact. This inspired some participants to question their identity. For example, Jane (O) described how she could no longer multitask, which impacted her sense of self:

I found it quite disconcerting, having been someone who was very organised to then find that I couldn’t achieve it. Something that you were good at is taken away from you … its quite an odd feeling (Jane).

Likewise, Margaret (O) said:

I was really upset by it, and I was in tears because I couldn’t do my job … It meant a lot to me in terms of my self-worth and self-belief in terms of my job and how I deliver my work because all of a sudden, I can’t (Margaret).

Similarly, the younger women also described how they no longer felt themselves and often questioned their self-belief in terms of their intelligence. Both Dolly (Y) and Penelope (Y) expressed similar threats to their idea of who there were:

I feel stupid. I’m not stupid, but it’s coming out that way (Dolly).

I don’t feel as switched on … makes me feel a bit thick (Penelope).

Similarly, when discussing taking control, the older women commonly highlighted how taking HRT enabled them to feel more themselves. For example, Melissa (O) shared the same experience:

You feel a bit more in control. You feel a bit more on it (Melissa).

Although this wasn’t always the case as taking HRT was also seen as a sign of no longer being young. As Caterina (O) said:

HRT makes you feel really old … but I’m OK taking it if it helps and allows you to lead a good life (Catarina).

## Discussion

The present study explored the experiences of brain fog within two different female age groups at either end of their reproductive journey. The analysis described three themes and a transcending theme.

The first theme addressed the ‘Daily disruptions of brain fog’, highlighting how brain fog was experienced as a form of cognitive dysfunction which could be triggered by a change in hormones or a lack of sleep. This is in line with the conceptualisation of brain fog in research by Ross et al. (2013) and Callan et al. (2022). It also reflects Greendale et al.’s (2009, 2010, 2020) model that brain fog’s aetiology is linked to hormones and the menopause. The results from the present study, however, also indicate that brain fog can be present in non-menopausal younger women who similarly described how their brain fog could be triggered by a lack of sleep or a change in hormones, through the contraceptive pill or their menstrual cycle. This is a novel insight, illustrating how brain fog is not limited to older women, or those who have a comorbidity, and may exist across the reproductive journey rather than just at the end.

The second theme focused on the ‘Cycle of impact’ and the emotional responses to brain fog. For many women, the stress of not being able to think clearly caused further anxiety making their brain fog worse with the younger women, in particular, worrying that their brain fog was a permanent change that would get worse as they got older. This reflects previous research highlighting the detrimental impact of brain fog on quality of life (Asadi-Pooya et al., 2022; Callan et al., 2022). It also illustrates how these negative emotions created a cycle of impact whereby feeling of stress and anxiety about brain fog could make the symptoms of brain fog worse. This reflects much recent research on the use of Cognitive Behaviour Therapy (CBT) and mindfulness-based interventions as a means to manage menopause symptoms (Hunter and Smith, 2021). Not all emotional responses to brain fog were negative, however, and some of the older females also felt positive emotions, including feelings of acceptance, and some described actively managing their emotions to reframe them in a positive way to avoid the cycle of impact. Much previous research has highlighted a role for positive framing described within the context of adaptation, acceptance or benefit finding and the use of Acceptance Commitment Therapy (ACT) for a range of chronic conditions such as cancer, chronic fatigue, rheumatoid arthritis, irritable bowel syndrome, Inflammatory Bowel Disease (IBD) and chronic pain (A-Tjak et al., 2015; Fernandes and McIntyre, 2020; Geenen and Dures, 2019; Giusti et al., 2021; Gray and Rutter, 2007; Matini and Ogden, 2016; Westbrook et al., 2019). The results from the present study indicate that whilst the younger women were more concerned by their brain fog, some of the older women were able to draw upon their life experiences and those of others around them to minimise the cycle of impact and even draw some benefit from their experiences.

The third theme focused on the participants’ practices of ‘Taking control’ of their brain fog, with all females reporting using task management strategies and methods of self-care such as sleep, diet and exercise. In addition, the older women also described being kind to themselves and some also using HRT. These findings reflect research highlighting the benefits of self-care methods such as diet and sleep to reducing brain fog (e.g. Shrividya and Joy, 2021). They also provide subjective support for the benefits of HRT even though the trial data remains inconclusive in terms of cognitive function (Langer et al., 2021). Further, they reflect some of the behaviour change initiatives recommended as part of CBT to manage menopausal symptoms (Hunter and Smith, 2021). Interestingly, they also suggest that whilst taking the contraceptive pill (to add hormones) can be a trigger to brain fog, taking HRT (to replace hormones) can help manage it.

Participants therefore described their experiences of brain fog in terms of daily disruptions, a cycle of impact and the ways in which they tried take control. Transcending these themes was the notion of a ‘Crisis of identity’, whereby all females questioned whether they were the same individual as they were before experiencing brain fog. In particular, the inability to perform tasks that they were once proficient at, evoked feelings of frustration and disconnect from their past capabilities, combined with uncertainty regarding the future of their cognitive abilities. This sense of crisis was apparent across all participants but seemed less impactful for some of the older women who were able to show a level of acceptance and resilience due to their own previous life experiences and those of the women around them. These more positive experiences of some of the older women reflect research exploring a range of chronic conditions highlighting sense making and adaptation drawing upon models such as the Self-regulatory model (Leventhal et al., 1980) and Cognitive adaptation theory (Taylor, 1983). In particular, the results highlight the ways in which individuals form schema about their physical symptoms as a means to cope and manage them. In contrast, however, notions of identity crisis reflect Bury’s (1982) term ‘biographical disruption’ which is used in sociological approaches to illness and the work of Charmaz (1983), who argued that patients with chronic illnesses experience a ‘loss of self’. In particular, having brain fog challenged these women’s sense of self and reflected a disjunction between who they were and who they are now. These findings also reflect the Self Discrepancy Theory (Higgins, 1987) which describes how crisis can occur due to a discrepancy a sense of one’s ideal self, current self and how others see us. In the context of brain fog, the discrepancy between who people were before and after developing brain fog together with the gap between how they see themselves now and how they would like others to see them creates a disruption in identity which can be anxiety provoking and uncomfortable. Perhaps, some older women experience less crisis as their sense of self before the onset of brain fog is more established due to the time spent as this person. Further, the knowledge that menopause is a transitional stage together with experiences of peers who have emerged the other side may act as protective factors. By comparison, young women may find brain fog more of a challenge if their identity is less established.

In summary, the findings from the present study indicate how, across their reproductive journey, both younger and older women have similar experiences of brain fog which can be triggered by a lack of sleep and hormone changes, exacerbated by worry and ameliorated through self-care, the use of task management strategies and hormone replacement when appropriate. The findings, however, also illustrate how brain fog can be so much more than episodes of cognitive dysfunction but can challenge a woman’s sense of self and make them question their identity as they compare their past capabilities with the distress of their current and potentially future brain fog. This sense of crisis may be more concerning for younger women if their identities are less established and less challenging for older women if their identities are less open to being disrupted.

### Methodological limitations

There are some limitations with this study, however, that need to be considered. Primarily the study recruited those reporting brain fog. Therefore, whilst the analysis of these interviews illustrated the negative impact of their experiences and consequences for their sense of identity it is possible that there are many who may experience brain fog but do not consider it an issue and therefore didn’t sign up for the study. Further, some may even experience aspects of cognitive dysfunction but do not use the label ‘brain fog’. Our findings therefore reflect the experiences of those who use this term and consider their experiences worthy of discussion. Second, we recruited two contrasting age groups to provide insights into the experience of brain fog across the reproductive journey. Whilst many similarities between these two sets of women were clear there were also some differences. Due to the qualitative nature of the study and sampling methods used, however, any comparisons should be considered tentative. Finally, all interviews were carried out by a woman from the younger age group which may have influenced how open participants from the two age categories felt able to be.

These findings have implications for research and practice. In terms of research, whilst current assessment tools focus on the cognitive dysfunction aspects of brain fog (e.g. Lynch et al., 2022; Mitchell, 2013; Smith et al., 2007) such tools could be broadened to incorporate the more psychosocial components of this condition. Furthermore, research could address the experiences of brain fog and whether they vary not only by age or stage of reproductive journey but also by culture and the meaning attached to different life stages and health conditions. In addition, the results from the present study indicate that whilst adding hormones (the contraceptive pill) can exacerbate brain fog in young women, replacing hormones (through HRT) can ameliorate symptoms in those women going through the menopause. Further research is required to assess the validity of this finding and what the mechanisms of this effect could be. In terms of practice, the results from the present study indicate that whilst many women find methods to manage their brain fog using strategies such as tasks management, being kind to themselves, diet and sleep some may need additional support to minimise the cycle of impact and any resulting crisis of identity. The results from the present study could therefore help inform the development of interventions to support women experiencing brain fog by focusing on the more psychosocial aspects of this condition. Such interventions could be tailored to the woman’s age and reproductive stage and whether they need support to develop better self care, improve their ability to find benefit or to focus on protecting or enhancing their sense of identity. Such interventions could be based upon existing protocols of CBT or ACT but also incorporate aspects of biographical disruption and self discrepancy theory depending on the experiences and needs of the individual.

To conclude, whilst previous research mostly conceptualises brain fog as a biological problem resulting either from hormonal changes or health conditions such as PoTS or Long Covid, the results from the present study suggest that it is much more than just biologically induced cognitive dysfunction. In particular, the interviews illustrated how brain fog can cause daily disruptions which can result in a cycle of impact as experiences are exacerbated through associated negative emotions. Further, whilst many women take control through acts of self-care or taking HRT experiencing brain fog can result in a crisis of identity as they perceive a break in their sense of self from who they are now compared to how they used to be. In addition, whilst any identified differences across the reproductive journey can only be tentative at this stage, it may well be that brain fog has a greater impact on younger rather than older women, if those who have a stronger history of not having brain fog can use this to buffer their sense of self against any challenges to their identity. Brain fog therefore may well be influenced by hormones, but it is experienced as a psychosocial phenomenon. Future research could develop measurement tools to assess a broader notion of this problem together with broader interventions to support women with the psychosocial aspects of this condition.
